# Locomotory Behavior of Water Striders with Amputated Legs

**DOI:** 10.3390/biomimetics8070524

**Published:** 2023-11-04

**Authors:** Javad Meshkani, Hamed Rajabi, Alexander Kovalev, Stanislav N. Gorb

**Affiliations:** 1 Functional Morphology and Biomechanics, Institute of Zoology, Kiel University, 24118 Kiel, Germany; 2 Division of Mechanical Engineering and Design, School of Engineering, London South Bank University, London SE1 0AA, UK

**Keywords:** locomotion, aquatic bugs, robotics, morphology, insects

## Abstract

The stability of the body during locomotion is a fundamental requirement for walking animals. The mechanisms that coordinate leg movement patterns are even more complex at water–air interfaces. Water striders are agile creatures on the water surface, but they can be vulnerable to leg damage, which can impair their movement. One can assume the presence of certain compensatory biomechanical factors that are involved in the maintenance of postural balance lost after an amputation. Here, we studied changes in load distribution among the legs and assessed the effects of amputation on the locomotory behavior and postural defects that may increase the risk of locomotion failure. Apparently, amputees recover a stable posture by applying leg position modifications (e.g., widening the stance) and by load redistribution to the remaining legs. Water striders showed steering failure after amputation in all cases. Amputations affected locomotion by (1) altering motion features (e.g., shorter swing duration of midlegs), (2) functional constraints on legs, (3) shorter travelled distances, and (4) stronger deviations in the locomotion path. The legs functionally interact with each other, and removal of one leg has detrimental effects on the others. This research may assist the bioinspired design of aquatic robots.

## 1. Introduction

The legs serve as supports for the body of insects in standing posture and during locomotion [1]. Stability of the body is an imperative requirement that must be maintained by all six legs [2,3]. Even though many studies have documented how the mechanical characteristics of legs influence the locomotion in terrestrial insects [4,5,6,7], the biomechanics of locomotion and the requirements for stability in semi-aquatic insects have only been studied to a very limited extent.

**Figure 1:**
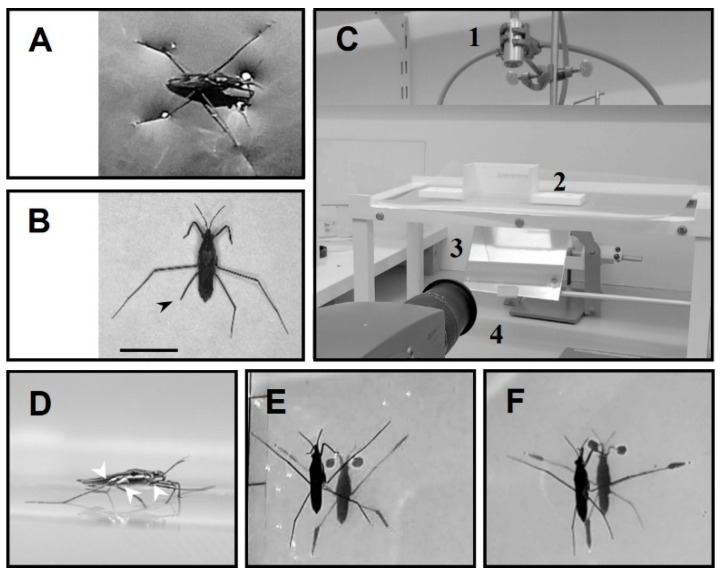
The experimental setup. (**A**) Water strider in its natural resting position. (**B**) Wild captured water strider missing part of hind leg (arrowhead). (**C**) The experimental setup including aquarium (2), light source on the top (1), high speed camera, (4) and mirror (3) below the aquarium. (**D**) Side view of a water strider in standing position, the white arrowheads indicate joints that were disabled by gluing. (**E**) Water strider during standing position while all legs are in contact with water surface. (**F**) Water strider with an amputation in the right hindleg. Scale bar = 5 mm.

Water striders are carnivorous insects that dwell on the calm surface of diverse waterbodies [8,9,10,11] (Figure 1A). Their body weight is supported by the water surface owing to the cohesive property of water molecules [12,13,14,15]. They perform striding, leaping and jumping to move around on the water surface for finding nutrients, chasing each other to mate or fight, and for predation avoidance [16,17,18,19,20,21]. It is common for water striders to be attacked by predators, such as birds, fishes and aquatic beetles [22,23,24], which may lead to physical injury with leg loss, as we frequently observed in the studied population of water striders (Figure 1B).

The effect of amputation on terrestrial locomotion has been studied extensively in insects with tripod gaits [25,26,27,28]. In view of their sculling abilities and sliding on the water surface, water striders are particularly suitable semi-aquatic insects to study the compensatory behavior during aquatic locomotion in amputated animals. It has been shown that water striders can modify their motion by regulating leg movements [29,30,31,32], but it is unclear, which compensatory leg movements facilitate their ability to cope with the new conditions associated with missing supporting legs. In addition, the efficiency of striding changes following leg amputations is unknown. Non-synchronized leg movements can reduce the efficiency of locomotion in insects due to the unbalanced body [33]. Earlier studies indicated that amputation in crickets leads to impairments of locomotory behavior [34], but this effect in a similar situation has not been documented in water walking insects. Water striders with their various body alignments are, therefore, suitable models to study the potential impairments of aquatic locomotion.

It has been not previously documented how water striders enact adaptive striding patterns when they become deprived of their natural leg number. In the present paper, we manipulated the legs by immobilizing them in order to investigate the effects of mechanical dysfunction on the insect’s mobility. Therefore, the severity of amputations was categorized in three grades based on their stable balance during standing and motion. Striding is more likely to be performed by amputees with one unilaterally or maximum two contralaterally amputated legs. But for individuals with bilaterally and ipsilaterally amputated legs, it was difficult or impossible to traverse the water surface.

Additionally, we conducted tests to investigate how the reduced number of supporting sites could affect the distribution of the bodyweight. As a means of understanding motion modifications after amputation, we assessed the alterations in body posture, as well as the sequential order of leg movements involved in performing a sculling stroke. This modelling approach may be useful in determining the biomechanical requirements for maintaining floating bodies on the water surface under a variety of conditions. In general, this research not only helps understand aquatic locomotion control in water striders but may potentially assist the bioinspired design of aquatic robots.

## 2. Material and Methods

### 2.1. Animals

Water striders, *Gerris argentatus*, were collected from a pond located in the botanical garden of Kiel University, Kiel, Germany. Several groups of amputees were established in the laboratory to simulate physical injuries. We immobilized their legs rather than cut them during the experiments to avoid wounds and causing misbalance by asymmetrical removal of weight. A bead volume of glue was applied to the joint between the coxa and femur while the femur was flexed upward to keep the tip of the target leg away from the water surface (Figure 1D,F).

### 2.2. Protocol of Measurement of Load Changes

Load change on the legs were estimated by the shadow tracking method, which has been used in previous studies [31,35,36,37]. As water striders float on the top of water, the surface below the legs is deformed in significant relationship with the load on each leg [38] (Figure 1A). As light beams pass through water, dimples cause them to be distorted, which leads to the appearance of a shadow area with a bright perimeter on the bottom of the aquarium. After proper calibration, the loads on individual legs can be estimated by measuring the size of these shadows [36,37]. According to the shadow method, vertical forces on the water surface have a significant correlation with the shadow area of the corresponding leg at the bottom of the vessel, and the bodyweight of water striders can be estimated by the total shadow areas of the legs [37].

Based on the measurements of leg shadows, the load applied by each leg can be expressed as a fraction of the total in percentage. In the present paper, based on the size of leg shadows, the load applied by each leg was calculated, and the measurements were converted into percentages to visualize the patterns of load changes for the amputated water striders.

### 2.3. Experimental Setup

Individuals were tested in a vessel with dimensions 10 cm × 5 cm × 5 cm (L × W × H) and filled with distilled water (ca. 5 mm in height), (Figure 1C). The tested groups performed striding freely, unassisted with external stimuli, to start or finish the motion. A source of illumination (*Storz Techno Light 270* Cold *Light* Projector, KARL STORZ SE & Co., Tuttlingen, Germany) was installed on the top. The bottom of the vessel was lined with a 125-micron white semi-transparent polymer sheet (GBC) to make the shadows visible. An inclined mirror (45 ) was placed below the vessel, to guide the shadow images to the camera. The animals were video-recorded over a period of 255 ms using a high-speed camera at 2000 frames per second (Olympus I-Speed 3 Series High-Speed Cameras, Olympus, Tokyo, Japan). The experiments were conducted at room temperature (25 °C).

### 2.4. Analysis Procedure

The frames of the captured videos were analysed using ImageJ to measure the shadow areas caused by the legs [39]. From the shadows, we tracked the positions of the body center and legs using the Manual Tracking plugin in ImageJ. We assumed the body center corresponded to the likely position of the center of body mass. Statistical analyses were performed by using SigmaPlot 12.0 (Systat Software Inc., San José, CA, USA).

### 2.5. Labeling of Legs and Their Amputations

**Figure 2:**
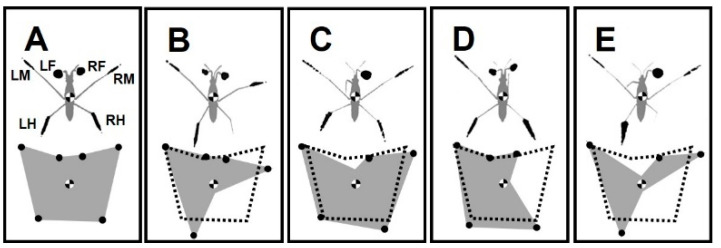
Illustration of shadow areas of legs and BOS for intact water striders and those with amputations. (**A**–**E**) The scheme of corresponding shadow area for the forelegs, midlegs and hindlegs which are labeled as LF, LM and LH, and as RF, RM and RH, on left and right sides, respectively. The black dots denote the contact points of legs. The scheme of BOS for the amputees. The normal BOS (shown with the dotted-line) is repeated in (**B**–**E**). (**A**) Intact water strider. (**B**) Right-hindleg amputation +/− RH. (**C**) Left-foreleg amputation −/+ LF. (**D**) Right-hindleg and left-foreleg amputation +/− RH & −/+ LF. (**E**) Right-midleg amputation +/− RM.

The dark spots on the bottom of the aquarium are the shadows of the legs (Figure 1E). The shadows correspond to the dimples in the water surface tension film under the legs. The shadows of forelegs, midlegs, and hindlegs on the left and right sides are indicated by (LF, LM and LH) and (RF, RM and RH), respectively [40] (Figure 2A). FL, ML, and HL indicate the pairs of forelegs, midlegs, and hindlegs, respectively.

We amputated animals by immobilizing the target legs to assess different effects depending on the disability of particular legs or their combinations. “−” indicates the body side with the amputated leg, “+” represents the normal side of body, and “&” indicates the combination of two amputations. This study addresses the impact of unilateral (−/+ or +/−), bilateral (−/−), ipsilateral (−/+ & −/+) and contralateral (−/+ & +/−) amputations. Accordingly, the amputation possibilities are listed in Table 1.

## 3. Results

### 3.1. Severity Grades of Amputations

**Figure 3:**
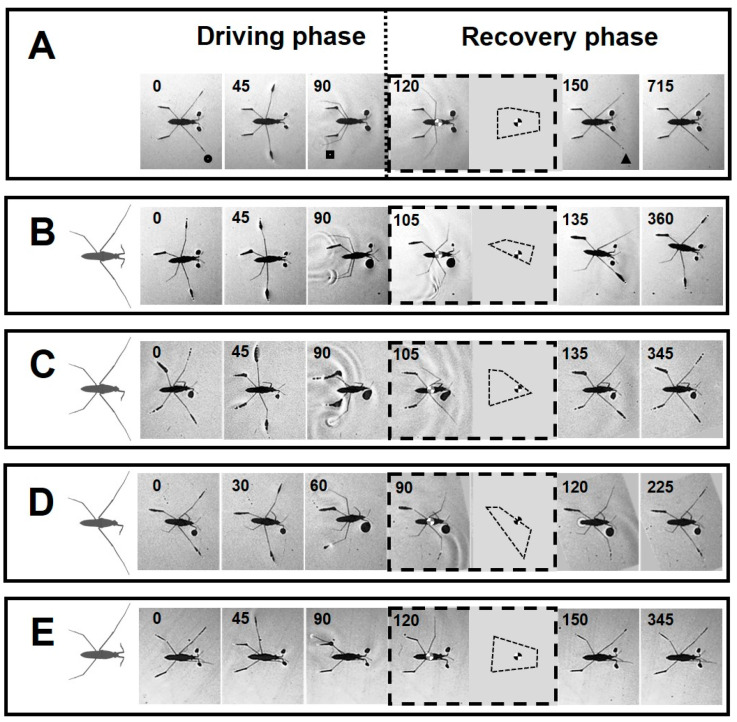
Sequences of leg kinematics during striding cycles. (**A**–**E**) The body position within a striding sequence for intact and individuals with +/−RH, −/+ LF, +/− RH & −/+ LF and +/− RM amputations, respectively. The insets indicate the BOS and the position of the center of the body during swinging the midlegs. The number in each frame indicates the time in millisecond. (**A**) Dotted line separates the driving phase (**left**) and the recovery phase (**right**). The circle, square and triangle indicate three key positions of the midlegs at the catch, finish and recovery positions, respectively. During the driving phase, the midlegs travel backward from the catch position to the finish position and touch-off from the water surface. The recovery phase starts with swing of the midlegs while the body passively continues sliding. During passive sliding, the body slides across the surface of the water without effort of the midlegs. The midlegs after swing touch-down to the water surface at the recovery position.

A common trait among all amputees was the inability to perform straightforward striding. Striding occurs through the symmetrical process of sculling, which is carried out by the midlegs (scull-legs) while the body slides on the water by the forelegs and hindlegs (ski-legs) [31,35] (Figure 3A). A sculling stroke is generated by the midlegs during the driving phase as the tips of legs move backward from the catch position to the finish position. Following this, the midlegs swing forward through the air to the recovery position, while the body continues to slide without effort during the passive sliding. On the basis of the experimental data, we classified the severity effect of amputations into three grades based on their ability to execute sculling (Table 2).

Grade I: Amputees, with ability to maintain standing position, can execute sculling (+/− RH, −/+ LF, and +/− RM) (Figure 2B,C,E and Figure 3B–E).

**Figure 4:**
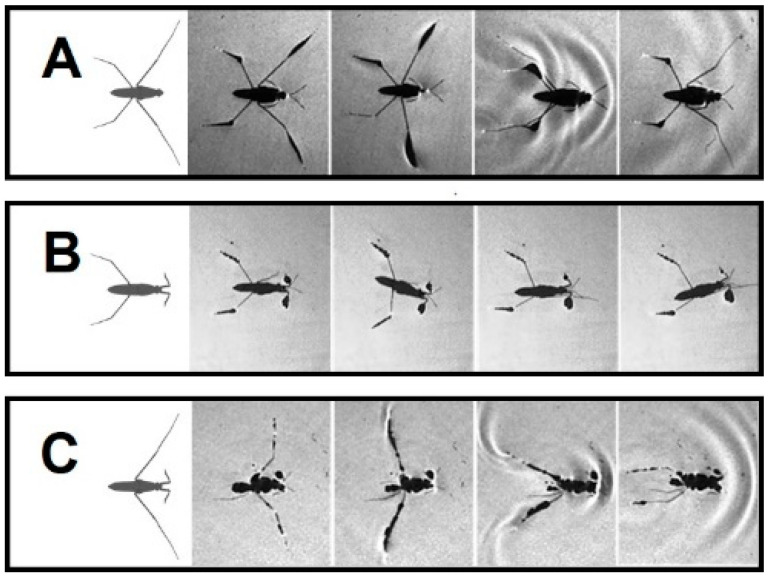
The body positions for water striders with bilateral amputation. (**A**) Forelegs amputation −/− FL. (**B**) Midlegs amputation −/− ML. (**C**) Hindlegs amputation −/− HL.

Grade II: Amputees can stand on water, while unable to perform a typical striding (+/− RH&−/+ LF and −/− FL) (Figure 2D, Figure 3D and Figure 4A). In −/− FL, the ventral side of the thorax serves as a bearing point for a short period of time during swinging of midlegs (Figure 4A). In +/− RH&−/+ LF, the right midleg never swings through the air and stays attached to the water surface.

**Figure 5:**
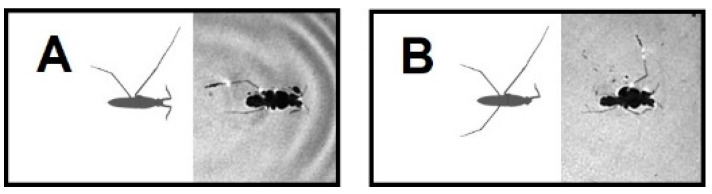
+/− HL & +/− ML (**A**) and +/− FL & +/− ML (**B**) amputations were trapped in water immediately after they were put on the water surface.

Grade III: This grade of amputation leads to the inability to stand and move over water (−/− ML, −/− HL, +/− RH & +/− RM and +/− RF & +/− RM). In this case, the water striders are trapped in the water and sink shortly after being above the water surface (Figure 4B,C; Figure 5A,B). Similar outcomes are expected for more severe amputations. Individuals without both midlegs can remain over the water surface and perform a staggering motion (Figure 4B). The motion is accomplished by rotation of the body to one side and pulling forward the hindleg on the other side, then repeating the motion to the other side. These amputees can only travel over a very short distance of a few millimeters. Although the grooming behavior is normal behavior for intact individuals, with of rubbing midlegs against forelegs or hindlegs on the same side, in our observations, +/− RH & +/− RM and +/−RF & +/− RM amputations were trapped by the water as soon as they were put on the water surface (Figure 5A,B).

Amputees of all types demonstrate non-symmetrical leg movement in comparison with intact individuals. In this study, we report on measurements mainly performed for amputees in Grades I and II.

### 3.2. Changes in Standing Posture

Generally, water striders stand with six points of contact, while the body center is located over the middle of the base of support (BOS) (Figure 2A). BOS refers to the imaginary area beneath the body and within the points where the legs contact the surface. BOS is associated with maintaining the equilibrium of body posture [41]. An amputation leads to an asymmetry in the BOS, while the area of BOS varies by the number of legs circumscribing the area. On the same scale, the BOS for the studied insects showed a variety of shapes and sizes (Figure 2B–E). BOS measurements for the intact, +/− RH, −/+ LF, +/− RH & −/+ LF, and +/− RM amputated individuals were 274, 177, 288, 144, and 220 mm^2^, respectively.

### 3.3. Load Change on the Legs during Locomotion

**Figure 6:**
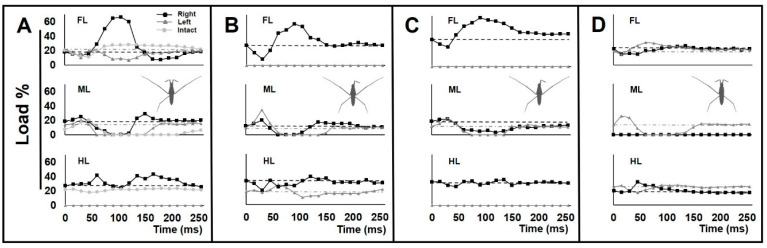
Load changes on the legs during striding. (**A**–**D**) The graphs of load changes on the legs in percentage for individuals with +/−RH, −/+ LF, +/− RH & −/+ LF and +/− RM amputations, respectively. Each graph shows the mean value of load, with the legs of each pair presented in one plot. Black lines with squared joints represent right-legs, and gray lines with triangled joints represent left-legs. The dashed lines are the baselines that represent the average load value applied on right-legs in the static state, and the dash-dotted gray lines are the baselines that represent the average load value applied on left-legs in the static state. (**A**) Faint gray lines with circled joints represent normal mean values of load for intact individuals for reference (Supplementary Figure S1). N = 3.

The kinematics of leg sequences for amputees differed from those of intact individuals (Figure 3A–E). We plotted the load changes on the legs for amputees during a given time (Figure 6A–D). The load change graphs for amputees showed different load patterns for all legs, compared with intact individuals (Supplementary Figure S1).

For +/−RH, an abnormal increase in the load on the right foreleg was observed. This was accompanied by a decrease in the load on the left foreleg (Figure 6A). For −/+ LF, the initial load reduction on the only foreleg was followed by a massive load increase to a peak value (Figure 6B). The increasing load acting on the forelegs could be due to anterior leaning of the body. In both types of +/−RH and −/+ LF amputations, the swing duration of the midlegs was shorter than in intact animals, particularly on the affected side of body.

In the case of the +/−RH & −/+ LF amputation, the range of load disruption for the legs was more extensive than in all other amputees (Figure 6C). Also, the right midleg acted as a support during the passive sliding phase and was constantly in contact with the water surface (Figure 3D). Following the driving phase, the load increased on the remaining foreleg.

In the case of +/−RM amputation, with a shortened swing period, the load on the only midleg was higher than normal (Figure 6D). There was an unusually high level of pressure on the left foreleg and hindleg during the sculling stroke.

### 3.4. Positioning of Bodies during Striding Cycle

**Figure 7:**
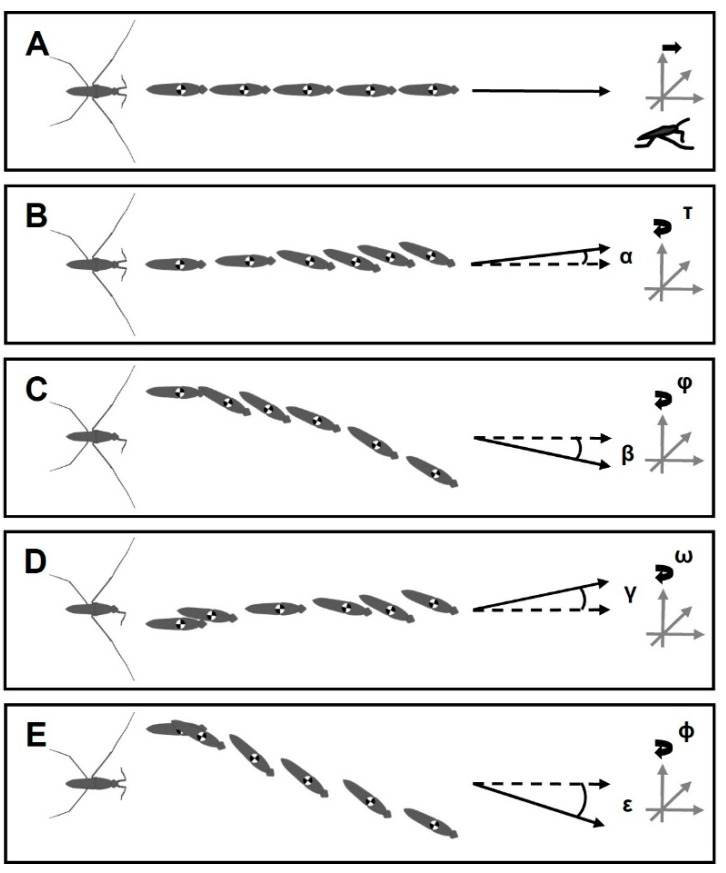
Illustration of the body trajectory. (**A**–**E**) The body positions during a striding cycle. The black arrow lines indicate the real direction of body sliding. (**B**–**E**) α = 8°, β = 21°, γ = 10° and ε = 47° show the angle of deviation between the direction of body sliding and the reference pose of the body (Dashed arrow lines). τ = 19°, ϕ = 29°, ω = 20° and φ = 35° and 9° indicate the spinning angles of heads relative to the vertical axis of the body center. (**A**) Intact water strider. (**B**) Right-hindleg amputation +/−HL. (**C**) Left-foreleg amputation −/+ FL. (**D**) Right-hindleg and left-foreleg amputation −/+ FL & +/− HL. (**E**) Right-midleg amputation +/− ML.

**Figure 8:**
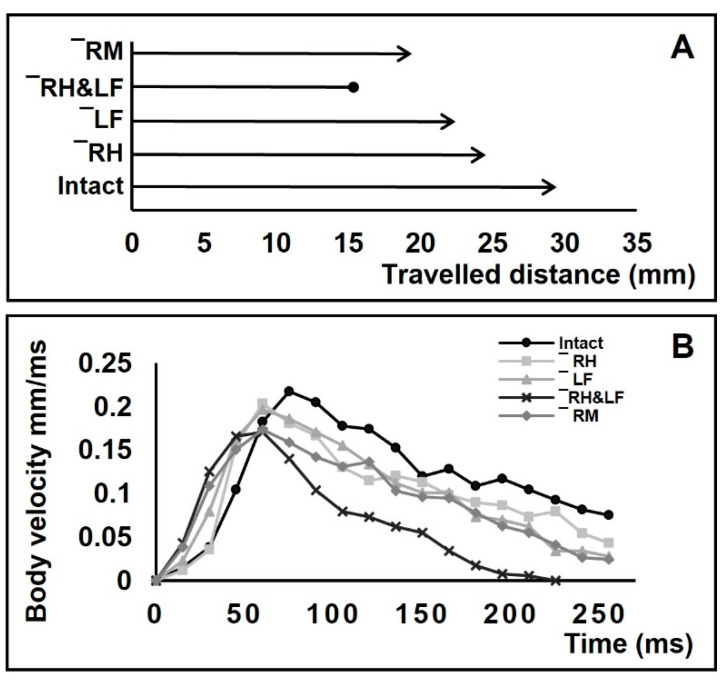
Comparison of travelling distance and the body velocity before and after amputations. (**A**,**B**) Travelling distances within a given time (255 ms) for intact, right-hindleg amputation +/− HL, left-foreleg amputation −/+ FL, right-hindleg and left-foreleg amputation −/+ FL & +/− HL, and right-midleg amputation +/− ML. The lines with arrowhead denote the bodies still moving, the line with a dot at the end denotes a body that stopped within the given time. (**B**) Velocity of bodies over time for intact, right-hindleg amputation +/− HL, left-foreleg amputation −/+ FL, right-hindleg and left-foreleg amputation −/+ FL & +/− HR, and right-midleg amputation +/− ML.

In amputees, the striding cycle was associated with heading error, while the bodies rotated around the vertical axis of the body center (Figure 7). Based on the body center tracking, the heading error angles for +/− RH, −/+ LF, +/− RH & −/+ LF and +/− RM were α = 8°, β = 21°, γ = 10° and ε = 47°, respectively, and the body yaw angles were τ = 19°, ϕ = 29°, ω = 20° and φ = 35° and 9°, respectively. For φ, we presented two values as the animals initially showed a sharp yaw angle, but the body continued sliding with a low change in the yaw angle. Contralateral amputation of one foreleg and one hindleg resulted in the rotation of the body in zigzagged patterns, but the striding cycle ended with a deviation, as well as rotation toward the direction of the amputated hindleg (Figure 7D). The amputees travelled shorter distances than intact animals during a given time (255 ms), with distances of 28, 23, 21, 14, and 18 mm for the intact, +/− RH, −/+ LF, +/− RH & −/+ LF, and +/− RM individuals, respectively (Figure 8A).

The loss of one foreleg and one hindleg, or contralateral limb loss, causes the velocity of the body to rapidly reach peaks that are lower than normal for intact individuals and then drop to zero in a short period of time (Figure 8B).

### 3.5. Jumping Ability

Although only striding has been characterised, the ability of the amputees to jump was also noted in this study. According to previous studies, synchronized movements of the midlegs and the hindlegs are required to perform jumping [30,32]. The amputee water striders with one missing foreleg, one hindleg, one midleg, both forelegs, a combination of one foreleg and one hindleg, and a combination of one midleg and one foreleg were able to jump, whereas individuals missing both midlegs, both hindlegs or a combination of one midleg and one hindleg were unable to jump. Consequently, water striders must possess a minimum of three middle and hind legs (both midlegs with one hindleg, or one midleg with both hindlegs) to be able to jump.

## 4. Discussion

### 4.1. Postural Change after Amputation

A highly sprawled position of the legs can provide insects with a stable posture [42]. However, insects can stand on fewer than six legs in contact with the water surface during the grooming behavior [43,44]. The absence of support from a single leg leads to immediate body postural changes [45]. Nevertheless, amputated insects adjust the legs to widen their stance in order to increase the size of the BOS [26]. As we observed for the examined amputee groups of water striders, the BOS changed in different ways due to different sets of weight-supporting legs (Table 2). With a larger area BOS, there is more chance for the center of the body to be positioned within the BOS and to increase body stability [28]. Based on the size and shape of the BOS, we anticipate that individuals are in unstable position in the following order with the first one as the most unstable: +/−RH&−/+ LF, +/− RM, +/− RH and −/+ LF (Figure 2B–E). Although the individuals without forelegs can perform a kind of sculling, those with an absence of support from the hindlegs are even unable to stay on the water surface (Figure 4A,C). Our previous study indicated that the midlegs play a compensatory role during absence of support from the forelegs [31]. Also, amputation of each hindleg alone or in combination with other legs has a more substantial impact on falling risk due to the strong shrinkage of the BOS. Based on the natural configuration of the legs, the removal of both hindlegs causes the center of the body mass to be located outside the BOS (Figure 2A and Figure 4C).

Compared with the pattern of weight distribution in intact individuals, the shadows below the legs of amputees resized, whereas the shadows on the left and right sides were not equal (Figure 2A–E). The leg sensory equipment assists with the control of load distribution among the legs [46,47]. Amputated insects can benefit from this mechanism to coordinate the rest of their legs with a gentle load shift among them. Quantifying changes in the shadow sizes of the legs provides a precise measurement that indicates the extra weight from removed legs was unevenly shifted to different remaining legs (Table 3). Based on the particular set of missing legs, the body shows some degree of leaning toward a side. In the absence of support from one leg, the adjacent leg, and the rest of legs on the same side of amputation primarily take the load bearing, and also those on the opposite side play a compensatory role to support the body.

The water striders are extremely efficient and agile water surface walkers, which makes them ideal for inspiring the design of robots that need to operate on water surfaces [48,49]. This knowledge can assist with understanding how multi-legged aquatic robots could coordinate support on the water surface to maintain a stable stance.

### 4.2. The Presence of All Legs Is Essential for Straightforward Striding

A major challenge for walking animals is maintaining body balance, particularly during the transition between two gaits, when the stability of the body is low [28]. A larger BOS that is obtained by widening the angle of the legs improves stability of the body during stepping [25,26,27]. In terrestrial insects, the BOS is small in size during the tripod gait when only three legs are in contact with the surface [3,28,42]. In addition to the shrinkage of the BOS, an amputation-induced condition moves the center of the body to the edge of the BOS, resulting in an unstable state, which is further exacerbated, when it is situated outside of the BOS [50,51,52,53,54] (Figure 2B–E). However, insects can slightly improve their mechanical efficiency by adjusting locomotory behavior after amputation [27].

Prior to performing motion, semi-aquatic insects can adjust the position of their legs, to achieve a six-legged starting posture with appropriate weight distribution [31,35,55]. In the case of severe types of amputation, falling of the body occurs in the standing position or immediately at the beginning of sculling (Figure 5). Despite this, some types of amputees can adjust their legs to execute striding. However, synchronization of midlegs movement, which is imperative for straightforward striding [56,57], is not commonly achieved for all amputees (Figure 3A–E).

Hence, the most important consequence of leg amputation was the change in the body locomotion trajectory (Figure 7). The body of disabled water striders rotated toward the affected side due to a lack of any support from the hindleg and midleg, or toward the opposite side after removal of the foreleg. This rotation causes the body to pull to the same side and results in the heading error.

A substantial risk of steering control loss can occur after the removal of a hindleg and particularly in combination with the amputation of a foreleg when the amputees were unable to keep the body in the initial posture (Figure 7B–D). On the other hand, following a sharp rotation about the vertical axis of body at the beginning of locomotion, the striding path was improved for the individuals with −/+ LF and +/− RM characteristics (Figure 7C,E). It seems that the hindlegs enable the insects to reverse the body rotation and direct it on a nearly straight path; however, a little heading error remains as the body continues sliding. The heading error is opposite or toward the side on which only the midleg or foreleg are in contact with the water surface in the cases of −/+ LF and +/− RM amputations, respectively. This shows that the hindlegs are functionally essential, but not sufficient, for steering.

In terrestrial insects, the legs work together to provide an optimal locomotion process [58]. Similarly, in water striders, all the legs are seen to play an essential role in efficient striding. Previous studies reported a rudder role for the hindlegs during striding [16,59,60]. However, the rudder function of hindlegs to direct and improve the locomotion trajectory is not independent of the natural movements of other legs. In other words, the hindlegs are unable to perform their steering function when other legs are not functioning properly. However, further studies are needed to uncover how the kinematic chain of each leg, which is dependent on the degrees of freedom of their joints, is important in motion trajectory control.

### 4.3. Asymmetrical Load Changes on the Legs during Locomotion

Sculling performance is well known to fluctuate with changes in body posture and the distribution of load on the legs [56,57,61]. During sculling, loading and unloading of the legs must occur symmetrically to ensure straightforward sliding [31,35]. Load shifting among the legs is an important factor strongly affecting the insect walking [50,62]. Insects are unable to execute stepping without the compensatory weight support by the other legs [45]. By switching from the more stable six-legged posture [1] to a stance with fewer legs, the body posture of the water strider becomes asymmetrical (Figure 2). In response, during striding, an irregular pattern is observed with increases and decreases in the load carried by the remaining legs that is different from the symmetrical pattern in an intact water strider (Figure 3 and Figure 6) (Supplementary Figure S1). This is important because the power generation for walking is influenced by the pattern of load distribution among the legs [63]. During passive sliding, the loads on the legs gradually return to the levels at the starting position. With dysfunction of the legs, the amputees are unable to avoid the irregularity of body sliding, and lean back to the normal posture, which causes a load perturbation on the legs (Figure 6). With an increasing number of amputated legs, the range of the load perturbation for the remaining legs was more extensive (Figure 6C). Thus, with the complete set of legs in intact animals which allows load balancing, the disturbance of locomotion performance remains minimal.

Amputation of any leg interferes with the load-bearing task of the other legs (Figure 2). Subsequently, disproportionate load distribution influences the sequential order of locomotion features, which leads to abnormality in the striding performance (Figure 3 and Figure 6 and Figure 7 and Figure 8). Earlier studies have shown that irregular loading of legs directly influences kinematics of insects [64,65]. However, during terrestrial locomotion, insects can coordinate the movements of their legs during stepping and modify their motion in response to load changes [66,67]. With a fair striding performance, individuals with +/− RH or −/+ LF can roughly re-establish their postural control. This shows that there is a degree of coordination between the legs which allows water striders to optimize their ability to float in water.

As a result of amputations, terrestrial insects change their stepping pattern due to alterations in their balance. In turn, this leads to an increasing energy cost of locomotion [34,68]. Water striders with amputations must put their legs in unusual positions to fulfil the supportive function, which can restrict their normal leg movements. Consequently, it becomes increasingly difficult for amputees to maintain their floating on the water surface. In addition, they are unable to achieve a smooth load shifting among the legs that is required for a gentle striding performance (Supplementary Figure S1).

### 4.4. Changes in Locomotory Behavior after Amputations

The horizontal thrust of body is exclusively provided through the sculling stroke, by using the midlegs [14,56,69,70]. In the present paper, this was also confirmed by observing the inability of the amputees without midlegs to execute striding (Figure 4B). Absence of support from one leg interferes with the kinematics of the other legs since the latter change their usual function, which in turn affects the efficiency of locomotion [45,71]. Quantification of velocity and travelling distance associated with the locomotion of intact water striders provided us with an indicator to assess the interaction between sculling and striding. Shorter traveling distances by amputees with only one midleg is attributed to impaired sculling stroke (Figure 8A). The travelling distances indicating the efficiency of sculling stroke were negatively affected by all types of amputation, even if both midlegs remained intact. It seems that missing any other supporting leg also leads to impaired locomotory behavior. Impaired locomotion also leads to reduced walking speed in terrestrial insects, such as cockroaches [72], mole crickets [73], and stick insects [58,74]. In water striders, the results are similar, showing that amputations affect both floating of the body and the generation of propulsion.

Despite having both midlegs, +/− RH & −/+ LF, +/− RH and −/+ LF amputees were found to be unable to execute symmetrical sculling. This exacerbates the lack of steering control that affects the locomotion parameters. For instance, the velocity of the body drops faster than normal (Figure 8B). The initial surge in body velocity that is followed by a gradual deceleration occurs during steady sliding [31]. The common feature of the amputees is a rapid drop in velocity from the peak that is gained during the sculling stroke (Figure 8B). It is unclear whether the presence of all legs is essential for a gradual reduction in the body velocity or if animals behaviorally reduce it in response to weak steering control. The striding cycle ended during the given time for the +/−RH&−/+ LF amputees, but the remaining amputees were able to maintain velocity to cover further distances. Affected by a lack of stabilizing function provided by the hindlegs and forelegs, amputees may have greater loss of control over their stability and thus reduce their speed to avoid toppling (Figure 8B). Thus, it can be deduced that instability of the body negatively affects striding during both driving and passive sliding phases. It shows the efficient striding of water striders requires synergic function of all legs, especially the midlegs. If missing any leg, water striders are unable to sustain a proper balance, and a considerable amount of propelling power may be wasted in attempting to maintain body floating and steering. This is manifested as a reduction in travelling distances during a given period of time (Figure 8A). Even so, the exact mechanism of how water striders control their speed during passive sliding is unknown, which can be explored in future studies. We predict the presence of certain mechanical constraints on the degrees of freedom in leg joints leading to unbalanced posture of the body that in turn increases the expenditure of energy.

### 4.5. Characterizations of Striding after Amputation

Utilizing their sensory system, insects govern their normal leg kinematics and adapt to various walking surfaces [45,75]. Adaptive spatiotemporal coordination patterns after leg amputation are known in cockroaches [45,68], stick insects [53,58], desert ants [76,77], and fruit flies [78,79]. Even after amputation, insects can execute a coordinated approach to adaptation of leg movement patterns to improve their mechanical efficiency [27]. In water striders, the leg pairs on two sides of the body normally move in synchrony with each other during both phases of driving and passive sliding. The supportive role of the legs extensively changes after amputation, mainly with asymmetrical alterations in leg movement timings (Figure 3). Amputations induce dramatic changes in patterns of the midleg movements, particularly in the timing of key events including sculling stroke, touch-off from and touch-down to the water surface, swing and so on (Figure 3). Water striders use asymmetrical sculling in some cases, such as when carrying prey. They support their bodies with one midleg and propel themselves forward with the other [80]. In the amputated water striders, the midlegs never detach from the water surface, or swing quickly in a shorter period of time (Figure 3). This illustrates that the midlegs are required to take more weight-bearing responsibilities as the body is in an instable state. It is a sign of coordination of the legs, which ensures a proportionate load distribution between the remaining legs. The assessment of the leg loading patterns of water striders indicates their partial dependency on each other. Although it seems that the sculling movements of midlegs are largely independent of each other, their kinematics are loosely coupled with the function of other legs (Figure 3).

An amputation impedes the natural leg placement and reduces the leg’s ability to govern movements. In fact, the dysfunction of each leg adversely affects the overall functioning of the locomotion system. Although their mobility continues, the amputees show difficulties in maintaining a stable locomotion trajectory, velocity and travelling distance (Figure 7 and Figure 8). However, amputated water striders can partially adapt the orchestration of leg movements to establish striding. Although the forelegs and hindlegs do not contribute to sculling, they may actively minimize the body instability imperfections to enable smooth locomotion [31]. There seems to be a predominant impact on striding performance associated with the loss of the hindleg (Figure 6 and Figure 7).

Amputations, depending on their severity, lead to behavioral changes in striding. Since the probability of falling becomes more pronounced, as the center of body approaches the edge of the BOS, animals reduce the time of sculling and sliding, depending on the severity of their amputation (Figure 3 and Figure 8). Hence, the present study represents a useful approach for understanding adaptability of the locomotory system of water striders to challenging situations.

The amputees, in some cases, can perform locomotion in a rather stable manner despite differences from the typical striding. Thus, water striders can be a good model system for the optimisation of walking robots after accidental damage. Our findings can also potentially help to develop aquatic-legged robots for use in environments with high risks of damage.

## 5. Conclusions

Water striders with missing legs achieve posture stability by adapting their BOS using their remaining legs. Despite not being sufficiently coordinated, they modify the position of their legs after amputation to improve weight distribution and avoid falling. Water striders spread their legs further apart from the body to compensate for the shrinkage of the BOS that results from a decreased number of contact points. Only individuals with one or two unpaired missing supports can stand over water, but this does not imply their ability to execute sculling. After amputation, the efficiency of striding becomes lower, the risk of falling higher, the travelling distance shorter and the maximum velocity lower. During locomotion, the amputees control leg movements by a fast recovery that is coupled with a rapid body re-alignment, which minimizes the induced irregularities in locomotion and prevents the body from toppling. There is a certain interdependence between the kinematics of each leg and that of the other legs. In either case, steering control error of the body appears to result from the loss of hindlegs and forelegs at the first and second ranks, respectively. Our results help to elucidate the adaptability of water strider locomotion to the challenging condition of missing extremities. Additionally, this research may facilitate the design of stable water-walking robots with different numbers of supporting limbs.

## Figures and Tables

**Table 1:** The amputation possibilities.

Symbols	Amputated Leg(s)
+/− RH	Right-hindleg
−/+ LF	Left-foreleg
−/− HL	Hindlegs pair
−/− ML	Midlegs pair
−/− FL	Forelegs pair
+/− RH & +/− RM	Right-hindleg and Right-midleg
+/− RF & +/− RM	Right-foreleg and Right-midleg

**Table 2:** Severity grades of amputations.

Grade I	Grade II	Grade III
+/− RH	+/− RH & −/+ LF	−/− HL
−/+ LF	−/− FL	−/− ML
+/− RM		+/− RH & +/− RM
		+/− RF & +/− RM

**Table 3:** Static measurements.

Animal Model	Load Value on Legs (%)						BOS (mm^2^)
	R1	R2	R3	L1	L2	L3	
Intact *	19	10	21	19	10	21	274
+/− RH	21	18	0	20	13	28	177
−/+ LF	30	10	22	0	11	27	288
+/− RH & −/+ LF	34	22	0	0	15	29	144
+/− RM	21	0	27	20	13	19	220

* Typical pattern of bodyweight distribution in intact water striders [31].

## Data Availability

All supporting data are made available in the article.

## Supplementary Materials

The following supporting information can be downloaded at: https://www.mdpi.com/article/10.3390/biomimetics8070524/s1. Figure S1: Load changes on the legs during striding. (**A**) The gray boxes in the background indicate the driving phase. The dashed lines are the baselines that represent the average load value applied on the legs in the static state. Gray lines are individuals, and the black lines are the mean for each leg. (**B**) The black lines are the mean for each leg pair. The dashed lines are the baselines that represent the average load value applied on the legs in the static state. Shadows below the forelegs, midlegs and hindlegs are labeled as LF, LM and LH, and as RF, RM and RH, on the left and right sides, respectively. FL ML and HL represent the pairs of forelegs, midlegs, and hindlegs respectively [31].
