# Supplementary material for: Locomotory Behavior of Water Striders with Amputated Legs
Source: Biomimetics (Basel). 2023 Nov 4;8(7):524. doi: 10.3390/biomimetics8070524 (PMC10669063; doi:10.3390/biomimetics8070524)
Supplement: Supplementary file 1 [file biomimetics-08-00524-s001.zip › biomimetics-2671732-supplementary.pdf]

Supplementary figures:

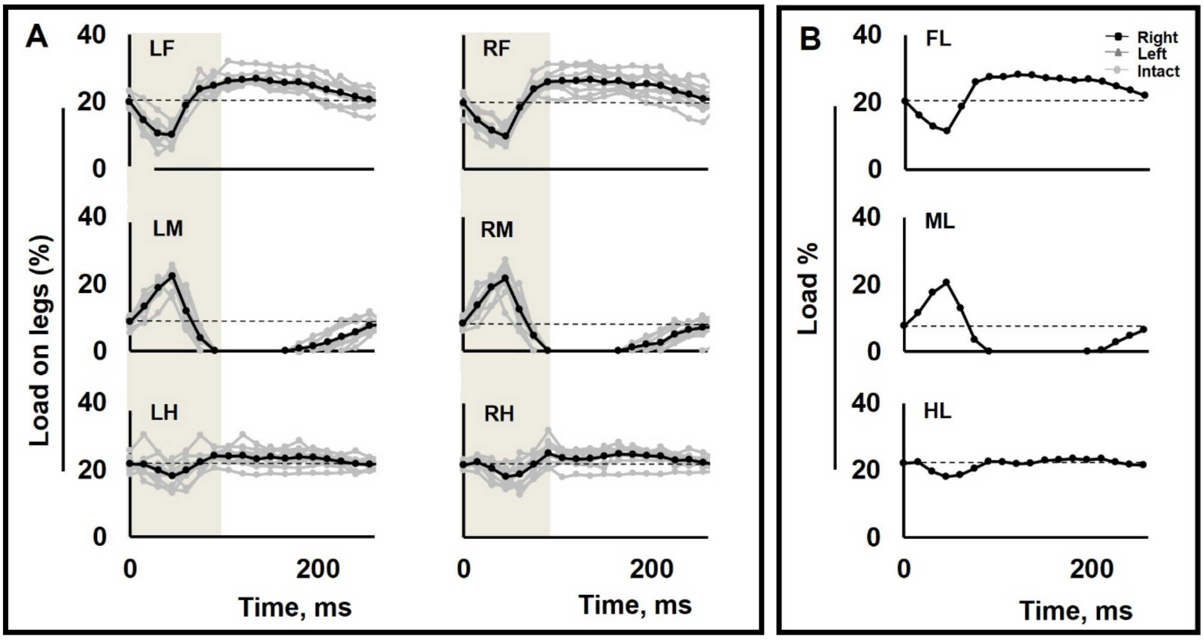

Figure S1. Load changes on the legs during striding. (**A**) The gray boxes in the background indicate the driving phase. The dashed lines are the baselines that represent the average of load value applied on the legs in the static state. Gray lines are individuals, and the black lines are the mean for each leg. (**B**) The black lines are the mean for each leg pair. The dashed lines are the baselines that represent the average of load value applied on the legs in the static state. Shadows below the forelegs, midlegs and hindlegs are labeled as LF, LM and LH on the left side, and RF, RM and RH on the right side, respectively. FL, ML and HL represent the pair of forelegs, midlegs, and hindlegs respectively [31].
